# Quantitative Analysis of Sex-Specific Feminizer (*fem*) Transcripts During Honey Bee (*Apis mellifera*) Development

**DOI:** 10.3390/ijms27062756

**Published:** 2026-03-18

**Authors:** Joanna Niedbalska-Tarnowska, Agnieszka Łaszkiewicz, Ajda Moškrič, Janez Prešern, Kinga Adamczyk-Węglarzy, Natalia Romek, Malgorzata Cebrat

**Affiliations:** 1 Laboratory of Molecular and Cellular Immunology, Ludwik Hirszfeld Institute of Immunology and Experimental Therapy Polish Academy of Sciences, Weigla 12, 531-114 Wroclaw, Poland; joanna.tarnowska@hirszfeld.pl (J.N.-T.); agnieszka.laszkiewicz@hirszfeld.pl (A.Ł.); kinga.adamczyk@hirszfeld.pl (K.A.-W.); natalia.romek@hirszfeld.pl (N.R.); 2 Animal Production Department, Agricultural Institute of Slovenia, Hacquetova Ulica 17, 1000 Ljubljana, Slovenia; ajda.moskric@kis.si (A.M.); janez.presern@kis.si (J.P.)

**Keywords:** *Apis mellifera*, feminizer (*fem*) gene, male- and female-specific transcripts, alternative splicing, sex determination pathway, quantitative PCR

## Abstract

Sex determination in honey bees (*Apis mellifera*) is controlled by the complementary sex determiner (*csd*) gene, which directs female- or male-specific splicing of the downstream feminizer (*fem*) transcript. Previous studies have reported contradictory data on the expression of *fem* transcripts in both sexes, but no rigorous quantitative analysis across developmental stages had been performed. Here, we optimized Real-Time PCR conditions to reliably detect and quantify both female-specific (*fem^F^*) and male-specific (*fem^M^*) transcripts, addressing challenges posed by AT-rich sequences, repeated regions, and cDNA instability. Using these methods, we analyzed transcript levels in eggs, larvae, and pupae of both sexes. Our results show that *fem^F^* is highly specific for females, with approximately 100-fold higher expression in females than in males, whereas *fem^M^* is less sex-specific, with only ~10-fold higher expression in males even at early developmental stages. Notably, *fem^F^* transcripts are detectable in males, and *fem^M^* expression increases in females during later pupal stages. Quantitative comparison indicates that *fem^M^* expression in males is similar to *fem^F^* expression in females, indicating that despite the presence of the premature stop codon in the male transcript, this transcript is not degraded through the mRNA surveillance mechanism. Our study provides a framework for evaluating *fem* transcript dynamics and has important implications for interpreting sex-determination mechanisms in honey bees.

## 1. Introduction

Sex in honey bees is determined by the allelic composition of a single locus called the complementary sex determiner (*csd*). Individuals that are heterozygous at the *csd* develop into females (either a queen or a worker), while haploids develop into males (they are hemizygous for *csd*) [1,2]. Individuals which are homozygous for *csd* can eventually develop into diploid males that form male reproductive organs but are sterile due to producing diploid sperm, and they are usually killed at the early stage of their development by nurse bees [3]. The zero fitness of the individuals carrying a homozygous *csd* genotype results in high mutational pressure, leading to the diversification of the *csd* gene; *csd* is highly polymorphic in honey bee species *Apis mellifera*, *A. cerana*, and *A. dorsata* [4,5,6,7,8,9,10,11,12]. The *csd* gene originated from a gene duplication event of the *fem/tra* progenitor gene. Comparative sequence analyses and phylogenetic studies have demonstrated [13,14] that *csd* and *fem* genes share extensive sequence homology, particularly within the SR-type protein domain, indicating a common ancestral origin. Following duplication, *csd* acquired novel allelic diversity localized in the newly established hypervariable region and evolved a sex-determining function, acting as the primary signal in the complementary sex determination system. Meanwhile, *fem* retained its role as the downstream effector.

**Figure 1:**
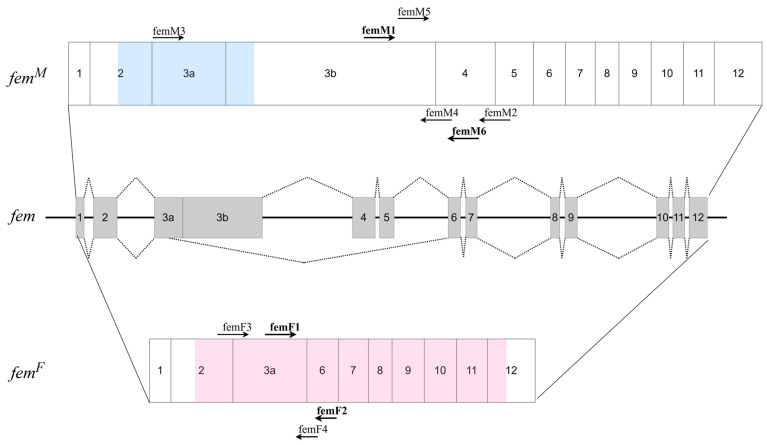
Genomic organization of the *fem* gene. Exons are shown as boxes and introns as connecting lines. The upper diagram represents the male transcript and the lower diagram represents the female transcript. The arrows indicate the positions of the primers used in the experiments. The blue and pink shadings represent the open reading frames (ORFs) of the male and female transcripts, respectively. Transcript structures are shown based on the reference sequences EU101389 (*fem^M^* mRNA) and EU101388 (*fem^F^* mRNA).

The prevailing set of evidence regarding the role of CSD protein in the sex-determination pathway shows that the presence of two different *csd* alleles in the embryo leads to female-specific splicing of the *fem* transcript consisting of exons 1/2/3a/6–12 (Figure 1). This results in the presence of a functional FEM protein, which, in turn, leads to female-specific splicing of the doublesex (*dsx*) transcript encoding a version of the protein truncated at the C-terminus. This initiates a cascade of female development. On the contrary, the lack of different *csd* alleles or a complete absence of *csd* gene products results in male-specific fem splicing (exons 1/2/3a/3b/4–12), encoding a non-functional protein due to the presence of a premature stop codon in exon 3b (Figure 1). The absence of the FEM protein leads to the appearance of a variant of the *dsx* transcript and protein that determines male development [1,2,15,16]. It has recently been shown that CSD proteins form trimers. The presence of the heterozygous *csd* genotype enables the formation of heterotrimers, in which CSD proteins interact with each other through their coiled-coil (CC) domains located immediately upstream of the RS-domain of the CSD. This type of CSD complex is thought to activate female-specific splicing of the *fem* transcript. The homozygous *csd* genotype manifests itself in the formation of CSD homotrimers, where CSD proteins interact through their identical (or nearly identical) C-terminal domains. These kinds of complexes are thought to be inactive in activating female-specific splicing of *fem* and lead to default male-specific splicing [16].

Interestingly, the scenario described above does not match the results obtained by another research group, which has shown that the female *fem* transcript is present in cells regardless of their *csd* status—i.e., whether they are heterozygous or homozygous for *csd*—while the male *fem* transcript is sex-specific and is present only in males [17]. This study suggests that the male FEM protein either directly promotes male development or acts as the dominant-negative regulator of the female FEM isoform. The experiment was performed in vitro using ectopic expression of *csd* genes and the *fem* minigene in a heterologous cell line (*Bombyx mori* BmN line); thus, direct comparison of these results and the findings of a study by Otte et al. [16], as well as the interpretation thereof, should be taken with some caution. However, their findings are supported by an earlier publication that also shows the expression of the female *fem* transcript in male embryos [18]. These earlier findings were not discussed in subsequent studies.

No rigorous quantitative analysis of the level of expression of sex-specific *fem* transcripts in distinct stages of bee development had been performed so far. The published results were obtained either at a single yet unknown time point under non-saturating PCR conditions [16] or after 35 cycles of PCR (presumably saturating conditions) [17]. Examination of the available literature also shows that amplification of sex-specific *fem* transcripts, in particular *fem^M^*, may have posed technical difficulties: in one of the studies the authors admitted that they excluded *fem^M^* from the analysis because it was infrequently and weakly detected in males [16], while another study inferred the presence of *fem^M^* from the presence of total *fem* transcript and absence of *fem^F^* [19].

In this study, we addressed the question of whether sex-specific fem transcripts are strictly restricted to a single sex or whether their relative abundance changes during honey bee development. We hypothesized that *fem* transcripts may not be completely sex-specific and that their quantitative ratios vary across developmental stages. Reliable testing of this hypothesis requires accurate and quantitative detection of both splice variants. Therefore, we first established experimental conditions allowing for robust qPCR amplification of both the female-specific (*fem^F^*) and male-specific (*fem^M^*) transcripts. After validating these conditions, we performed a systematic analysis of *fem* transcript expression across developmental stages and sexes to determine the relative abundance and developmental dynamics of *fem^F^* and *fem^M^* in *Apis mellifera*. By providing a quantitative framework for assessing the ratios of sex-specific fem transcripts, our study contributes to resolving discrepancies in the literature and to a more comprehensive understanding of the molecular mechanisms underlying sex determination in honey bees.

Our study provides (i) the first quantitative developmental profile of both transcripts from egg to pupal stages, (ii) efficiency-corrected qPCR quantification enabling comparison between transcript variants, and (iii) a quantitative evaluation of the relative abundance of *fem^F^* and *fem^M^* during development.

## 2. Results

### 2.1. Establishing a Method for Quantitation of Sex-Specific Feminizer Transcripts

To establish the conditions for Real-Time PCR quantification of sex-specific *fem* transcripts, we first focused on identifying suitable primer sets. Admittedly, this task is not trivial for the *fem* locus, as it contains repeated regions both within the *fem* gene itself and in the *csd* gene, which originated from a duplication event of *fem*. Moreover, the *fem* transcript sequence includes AT-rich, low-complexity regions, further complicating both primer design and optimization of reaction conditions. Additional criteria for primer selection were that the *fem^M^* and *fem^F^* amplicons should be of similar length (and not exceeding 200 bp) and located in separate exons to prevent genomic DNA amplification during the assay. To begin, we analyzed the primer sets used in previous studies: Otte et al. [16], Suzuki et al. [17], and Wang et al. [19]. The locations of these primers are shown in Figure 1. The primers used by Wang et al. [19] were excluded from our study because they were located within a single exon (specific for the male transcript). Primers specific for *fem^F^* used by Otte et al. [16] and Suzuki et al. [17] fulfilled our criteria, but primers for *fem^M^* produced excessively long amplicons (912 bp for femM3–femM4 primers and 458 bp for femM1–femM2 primers). We therefore designed new primers specific for the *fem^M^* transcript (femM5 and femM6; see Tables in Section 4.3 and Table S1) to be used with existing primers but yielding shorter products.

**Figure 2:**
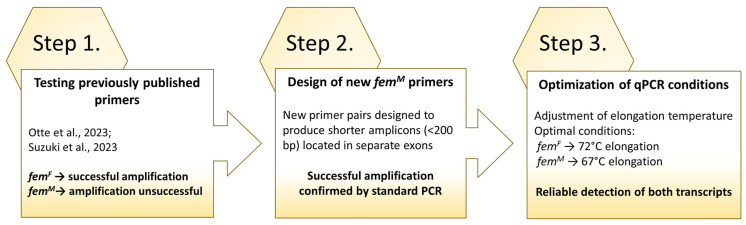
Optimization scheme of RT–PCR conditions for different *fem* transcripts, including testing of previously published primers [16,17].

The combinations of primers, reagents, and amplification conditions tested in this work are presented in Figure 2. Initially, we used commercial qPCR mastermixes, which unfortunately did not work with any of the primer sets. Consequently, we developed a custom master mix for Real-Time PCR to allow testing of different polymerases. The range of polymerases tested was limited due to our decision to include uracil-N-glycosylase and dUTP, aimed at eliminating carry-over contamination during the assays. The final composition of the reaction is provided in the Section 4. Using this mix, we successfully amplified the *fem^F^* transcript only with the femF1 ↔ femF2 primer set. Unfortunately, the initially tested primer combinations (femM3 ↔ femM4 and femM1 ↔ femM2) did not amplify the *fem^M^* transcript. We therefore extended the range of tested primer combinations and polymerases in standard PCR reactions. Eventually, we identified a working primer pair (femM1 and femM6; Tables in Section 4.3; Figure 2) in standard PCR; however, amplification failed when switching to Real-Time PCR. The key to solving this problem was the PCR conditions described in the study by Mukai et al. [20] recommending lowering the elongation temperature for amplification of AT-rich regions [20]. We found the 67 °C elongation temperature to be suitable for the amplification of *fem^M^* transcript. Finally, we verified the specificity of reactions by means of the cloning and sequencing of the amplification products.

In summary, we found that: (a) our custom Real-Time PCR mix worked for both *fem^F^* and *fem^M^* transcripts, (b) the optimal elongation temperatures were 72 °C for *fem^F^* and 67 °C for *fem^M^*, and (c) the primer pairs were of femF1 ↔ femF2 for *fem^F^* and femM1 and femM6 for *fem^M^*. Another important observation was that, in contrast to the housekeeping gene *rps5*, both *fem^F^* and *fem^M^* cDNA are highly unstable during freezing and thawing cycles, as indicated by constantly and significantly increasing C(t) values. Therefore, we strongly recommend using the cDNA template for *fem^F^* and *fem^M^* amplification immediately after synthesis.

**Figure 3:**
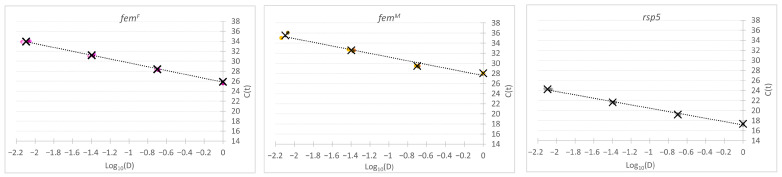
Standard curves used for validation of Real-Time PCR assays for *fem^F^* and *fem^M^*, and *rps5*. Serial fivefold dilutions of cDNA (1/1, 1/5, 1/25, and 1/125) were amplified under the optimized reaction conditions. Quantification cycle (C(t)) values were plotted against the logarithm of the dilution factor to generate standard curves for each target gene. Colored dots represent individual replicates, and X marks represent mean C(t) values obtained from at least three technical replicates. The curves were used to determine assay linearity, amplification efficiency, and coefficient of determination (R^2^).

To evaluate the performance of the primer sets selected for quantitative analyses, standard curves were generated for *fem^F^*, *fem^M^*, and the reference gene *rps5* using serial fivefold dilutions of cDNA (1/1, 1/5, 1/25, and 1/125). Each dilution point was analyzed in at least three technical replicates. Mean C(t) values and standard deviations were calculated for each point and used to construct linear regression curves (Figure 3, Table 1).

In accordance with the MIQE guidelines [21], assay performance was evaluated by determining amplification efficiency, linear dynamic range, and coefficient of determination (R^2^) for each primer set. All assays fulfilled MIQE criteria for quantitative Real-Time PCR, displaying linear amplification across the tested dilution range and efficiencies suitable for efficiency-corrected quantification. For all three assays (*fem^F^*, *fem^M^*, and *rps5*), amplification remained linear and precise across the tested dilution range. The lowest tested dilution (1/125) met the criteria for quantitative interpretation and was therefore considered the lower limit of quantification (LLOQ) of the assays. The efficiencies of all reactions were within the MIQE-recommended range, allowing for the use of three technical replicates in the assay (>80%). However, because amplification efficiencies differed among targets and were lower for the *fem* transcripts than for *rps5*, we decided not to use the simplified 2^−ΔΔC(t)^ method for relative expression analysis in subsequent experiments. Instead, each analysis included standard curves for every primer set.

To verify the suitability of *rps5* as a reference gene, its C(t) values were examined across all developmental stages and both sexes. The mean C(t) values showed limited variation (CV mostly below 4%), indicating stable expression of *rps5* and supporting its use for normalization (Table S2).

### 2.2. Expression Profile of Sex-Specific Fem Transcripts During Development

**Figure 4:**
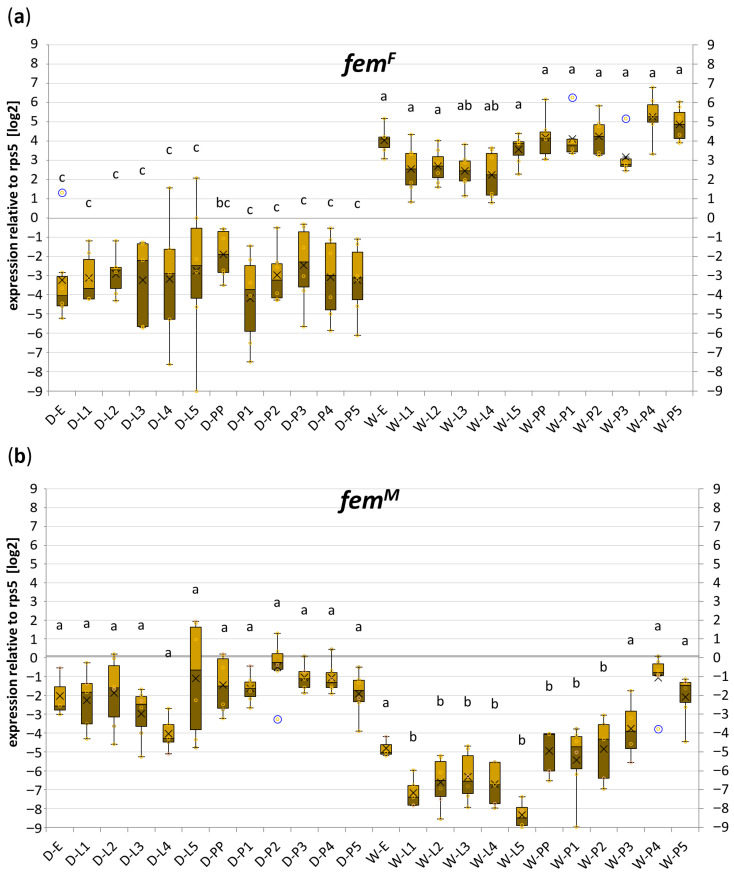
Developmental profile of *fem* mRNA expression. Box plots show the relative expression levels of *fem^F^* (**a**) and *fem^M^* (**b**) normalized to *rps5* (log_2_ scale) across different developmental stages of drones (D-) and worker bees (W-), including embryos (-E), larvae (-L1 to -L5), and pupae (-P1 to -P5). Individual values from biological and technical replicates are shown as light yellow circles, while blue circles indicate outliers. Statistical analysis was performed using the non-parametric Kruskal–Wallis test followed by pairwise Mann–Whitney post hoc tests. Identical letters above the boxes indicate groups that do not differ significantly (*p* < 0.01; *n* = 3 biological replicates).

After establishing the working conditions for *fem* transcript amplification, we conducted a quantitative analysis of the expression levels of sex-specific *fem* transcripts during female and male development. To achieve this, RNA was isolated from single specimens representing eggs (E), larval stages 1–5 (L 1–5), and pupal stages 1–5 (P 1–5). Following reverse transcription, the resulting cDNA was amplified under the optimized conditions described above. The expression levels of fem transcripts were normalized to the expression of the house-keeping gene *rps5*. The results of this analysis are presented in Figure 4a,b, while the list of mean C(t) values obtained for each transcript is presented in Appendix A, Table A1.

The obtained data showed a clear difference in the expression of *fem^F^* between females and males, regardless of the developmental stage (Figure 4a). The level of *fem^F^* expression remained constant throughout development within each sex, with a slight but statistically insignificant increase observed in the later stages of female development. The difference in *fem^F^* expression was approximately 100-fold higher in females than in males. In contrast, the difference in the expression level of the *fem^M^* transcript between sexes was less pronounced (Figure 4b), averaging around 10-fold higher expression in males. Moreover, *fem^M^* expression in females increased significantly across developmental stages, reaching in stages P3–P5 levels that were indistinguishable from those observed in males.

Employing qPCR analysis with quantification based on standard curves generated individually for each transcript and precisely quantifying the reaction efficiencies allowed us to determine not only the relative differences in the expression of each transcript between the sampled individuals, but also the differences in expression levels between the transcripts themselves. We have found that the expression of *fem^M^* in males did not significantly differ from the expression of *fem^F^* in females (approximately 2-fold less in males).

## 3. Discussion

While the general model that *fem^F^* is female-biased and *fem^M^* is male-biased has been established previously, the quantitative developmental dynamics of these transcripts have not been systematically analyzed.

Here, we provided results on quantitative expression values for *fem^F^* and *fem^M^* from different stages of honey bee development for drones and workers for the first time. The previously reported difficulties in amplifying and quantifying the *fem* transcript—specifically the *fem^M^* variant [16]—may have been attributed to its overall low expression level, even in males. This could result from the presence of a premature stop codon in exon 3b, which may trigger the nonsense-mediated decay (NMD) pathway. NMD is a widely conserved surveillance mechanism in eukaryotes whose primary function is to reduce the abundance of erroneous mRNAs containing premature termination codons. However, our results are consistent with the possibility that the *fem^M^* transcript is not efficiently degraded by the NMD pathway, since its expression level in males is not significantly lower than that of *fem^F^* (the form lacking a premature stop codon) in females. Direct experimental verification of this possibility would require targeted inhibition of the NMD pathway, for example by combining transcript analysis with NMD inhibitors such as cycloheximide or by applying complementary approaches such as Northern blotting. Our experience gained during the optimization of *fem* transcript amplification points instead to technical rather than biological causes for the previously reported issues. The *fem* transcripts are AT-rich, with AT content exceeding 70% in the amplified regions and peaking at up to 90% within 40-nucleotide windows. AT-rich sequences are known to form secondary structures that can impede polymerase progression [22] and are more prone to strand breakage [23]. We assume that issues related to cDNA stability and polymerase processivity become even more pronounced when amplifying relatively long amplicons, as reported previously [16]. Furthermore, when comparing the expression levels of two transcripts using qPCR, it is advantageous to use amplicons of similar length to avoid artefacts arising from differences in RNA or cDNA stability. The primer set for *fem^M^* that ultimately proved successful in our hands produced a 168 bp amplicon, closely matching the length of the *fem^F^* amplicon (177 bp), though with a higher AT content (78% vs. 71%). This difference in AT content may explain why *fem^M^* amplification was only possible at a lower extension temperature.

Our data on the expression profile of sex-specific *fem* transcripts generally corroborate the prevailing view that the *fem^F^* transcript is female-specific, as we observed its expression to be approximately 100-fold higher in females than in males. However, we consistently detected *fem^F^* transcripts in males, raising questions about the mechanism underlying *fem^F^* splicing in the absence of a heterozygous *csd* genotype. As shown in Appendix A, Table A1, the C(t) values for *fem^F^* transcripts in males averaged around 33 cycles, which may explain the detection of *fem^F^* transcripts in males (or cells homozygous for *csd*) reported by Suzuki [17], who used 35 amplification cycles, thereby exceeding this detection threshold. It is also possible that the level of *fem* expression from the transgenes used in that study was much higher than under natural conditions, rendering the 35-cycle point unsuitable for quantitative comparison. Our observations regarding the expression profile of *fem^M^* contradict those reported by Suzuki et al. [17], as we did not observe the lack of *fem^M^* in females. Moreover, the difference in *fem^M^* expression between females and males was considerably smaller (~10-fold) than that observed for *fem^F^*.

It is particularly interesting that the findings of Suzuki et al. [17] indicate that, in the absence of *csd*, the *fem* transcript is not processed into either the male- or female-specific form. According to their results, the presence of a single *csd* allele was sufficient to produce processed *fem* transcripts, suggesting that CSD homotrimers are functional and required for the formation of the male-specific *fem* transcript. This interpretation stands in contrast to the results of Gempe et al. [15], who reported that the absence of *csd* leads directly to male-specific *fem* splicing. However, those experiments relied on RNAi-mediated knockdown in female embryos, a method that may leave residual *csd* expression. If low levels of CSD protein were still present, homotrimers—assumed to form more readily than heterotrimers—could have assembled and, assuming such homotrimers are indeed functional, driven male-specific *fem* splicing. The possibility that CSD homotrimers actively generate male-specific *fem* transcripts may also help explain the relatively high expression of *fem^M^* we observed in females, as the heterozygous (female) *csd* genotype does not preclude the formation of CSD homotrimers in embryonic cells. Furthermore, the gradual decrease in *csd* expression during development could allow homotrimers to predominate at later stages [1], which is consistent with the increased *fem^M^* expression in female pupae observed in our study. It is possible that the presence of the male splice variant in females represents background splicing noise occurring when regulatory control becomes less stringent at later developmental stages, or that it reflects an as yet unidentified regulatory function of the male transcript. However, we believe that the interpretation of *fem* transcript expression should not be considered independently of CSD activity. The formation of different CSD trimer configurations provides a plausible mechanistic framework linking the observed expression patterns with the molecular regulation of sex-specific splicing. Admittedly, these conclusions remain highly speculative and require validation through rigorous double-allele *csd* knockout experiments, which would definitively clarify whether CSD homotrimers are capable of driving male-specific *fem* splicing in vivo.

Although the analyzed individuals were collected from several honey bee colonies and randomly selected for the experiments, the samples originated from a single regional population. Therefore, the observed expression patterns should be confirmed in future studies using honey bee populations from other geographic regions. Such comparative analyses could help determine whether the developmental dynamics of fem transcripts observed here represent a general feature of *Apis mellifera* biology.

In summary, our data demonstrate that sex-specific *fem* transcripts are not strictly limited to a given sex in honey bees. While *fem^F^* is highly specific for females, showing approximately 100-fold higher expression in females than in males, *fem^M^* is less sex-specific, with only about 10-fold higher expression in males than in females even at early developmental stages. These findings have important implications for interpreting experiments on sex-determination pathways and transgenic studies, emphasizing that accurate sex assignment based on *fem* expression should rely on quantitative methods, and that the utility of assessing *fem^M^* expression may be reduced in the later stages of development.

## 4. Materials and Methods

### 4.1. Sample Collection

Biological material was obtained from several honey bee colonies headed by naturally inseminated 1-year-old queens. Individuals at different developmental stages were identified based on morphological characteristics, sampled and placed in separate tubes, immediately immersed in TRIzol reagent, and stored at −80 °C. Samples were collected for eggs (E), larvae stages 1 to 5 (L 1–5), prepupae (PP), and pupae stages 1 to 5 (P 1–5).

### 4.2. RNA Isolation

Total RNA was isolated using the standard TRIzol method [24], applying reagent volume according to the sample type (E—250 µL, L1–L3—500 µL, L4–L5—1000 µL of TRIzol reagent) and scaling the volume of other reagents accordingly. For larval stages L4 and L5, half of the larval body was used for RNA extraction, while for pupae RNA was isolated separately from the thorax and the abdomen. RNA concentration and purity were measured using a NanoDrop One spectrophotometer (Thermo Fisher Scientific, Madison, WI, USA), and RNA integrity was verified by agarose gel electrophoresis. Up to 2 µg of RNA was used to synthesize cDNA using MultiScribe™ Reverse Transcriptase (Thermo Fisher Scientific Baltics UAB, Vilnius, Lithuania) according to the manufacturer’s instructions. The resulting cDNA was diluted twofold with ultrapure water and stored at −80 °C. Each developmental stage was represented by three independent biological samples. Individuals were randomly selected from a larger pool of samples originating from several honey bee colonies.

### 4.3. Real-Time RT-PCR

Table 2 lists all the oligonucleotide primers tested in this study. The primer pairs ultimately used for quantitative analysis are shown in bold. Throughout this study, numerous amplification conditions were tested, and the rationale for establishing the final workflow is presented in the Section 2. To maintain clarity, this section only describes the conditions that were found to be effective and used in the final analyses.

The following reagents were used to create a custom mix for Real-Time RT-PCR amplification: Taq polymerase buffer (1× concentration, Dream Taq buffer Thermo Fisher Scientific Baltics UAB, Vilnius, Lithuania), 0.5 µM of each primer, 0.2 mM dCTP, dGTP, dATP, 0.4 mM dUTP (Jena Bioscience GmbH, Jena, Germany), 0.004 U/µL uracil-N-glycosylase (Jena Bioscience GmbH, Jena, Germany), 0.5 µM EvaGreen (Jena Bioscience GmbH, Jena, Germany), 0.05 µM ROX (Jena Bioscience GmbH, Jena, Germany), 0.05 U/µL Taq polymerase (EURx Ltd. Gdańsk, Poland), and 1 µL of cDNA. The reaction was performed in a total volume of 10 µL.

Amplification was performed using QuantStudio 3 Real-Time PCR System (Thermo Fisher Scientific, Singapore) under the following cycling conditions (Table 3):

C(t) values were determined after establishing a constant threshold level for all genes and all replicates. For each biological sample included in the analysis, a corresponding no-reverse transcription control (RT−) was prepared and processed in parallel with the cDNA samples. RT− controls were subjected to Real-Time PCR alongside their matched cDNA counterparts using identical reaction conditions and primer sets. Data were analyzed using standard curves generated from single-template reactions and normalized to the reference gene *rps5*. Statistical analysis was performed using the non-parametric Kruskal–Wallis test followed by pairwise Mann–Whitney post hoc tests. For better visualization of group differences, data were log_2_-transformed prior to plotting.

### 4.4. Cloning and Sequencing of the Amplification Products

The amplification products were cloned into pJET 1.2 vector contained in the CloneJET PCR Cloning Kit (Thermo Fisher Scientific Baltics UAB, Vilnius, Lithuania) according to the manufacturer’s recommendations and having performed blunting of the amplicons prior to ligation. The ligation products were transformed into a chemicompetent bacterial strain of *E. coli.* Single colonies were picked and used directly in a PCR reaction (30 cycles, 52 °C annealing) using pJET1.2F and pJET1.2R primers. The amplification product was digested with alkaline phosphatase (0.25 U, Thermo Fisher Scientific Baltics UAB, Vilnius, Lithuania) and exonuclease I (0.5 U, Thermo Fisher Scientific Baltics UAB, Vilnius, Lithuania) for 30 min at 37 °C. The enzymes were inactivated by denaturation (5 min, 95 °C). One of the DNA was used in cycle sequencing reaction (10 µL) containing 1.9 µL 5× sequencing buffer, 0.5 µL BigDye Terminator Cycle Sequencing mix (Thermo Fisher Scientific Baltics UAB, Vilnius, Lithuania) and 0.65 µL 5 µM of either pJET1.2F or pJET1.2R primer. The sequencing products were purified using Sephadex G-50 columns, denatured and subjected to capillary electrophoresis (ABI Prism 310, PE Applied Biosystems, Foster City, CA, USA). Data were analyzed using ABI Sequence Analysis software (v3.3).

## Figures and Tables

**Table 1:** Parameters of Real-Time PCR standard curves for *fem^F^*, *fem^M^*, and *rps5*. Mean C(t) values and standard deviations were calculated for serial fivefold dilution points (1/1, 1/5, 1/25, and 1/125) based on at least three technical replicates. Amplification efficiency (E, %), regression equation, and coefficient of determination (R^2^) were derived from linear regression of the standard curves. Amplification efficiency and its 95% confidence interval (CI) were calculated from the slope using Student’s t distribution (*n* − 2 degrees of freedom) and propagated to efficiency.

	*fem* ^F^		*fem* ^M^		*rps* ^5^	
Primers	femF1 ↔ femF2		femM1 ↔ femM6		rsp51 ↔ rsp52	
Dilution (D)	mean C(t)	SD C(t)	mean C(t)	SD C(t)	mean C(t)	SD C(t)
1/1	25.88	0.18	28.05	0.06	17.35	0.12
1/5	28.41	0.14	29.49	0.08	19.22	0.09
1/25	31.20	0.12	32.63	0.12	21.67	0.10
1/125	33.93	0.11	35.55	0.54	24.27	0.07
	y = −386x + 24.65		y = −3.67x + 27.588		y = −3.32x + 17.143	
Slope	−3.86		−3.67		−3.32	
95% CI (slope)	−4.06–−3.65		−4.16–−3.17		−3.41–−3.24	
Efficiency [%]	82%		87%		99.90%	
95% CI (Efficiency)	76–88%		74–107%		96.39–103.75%	
R^2^	1.00		0.98		0.99	

**Table 2:** Primer sequences used for amplification of fem transcripts. Forward and reverse primer sequences used for amplification of *fem^F^*, *fem^M^*, and the reference gene *rps5*. Primer pairs shown in **bold** were successfully used under the described Real-Time PCR conditions and selected for the analysis of *fem* transcript expression in drones and worker bees across developmental stages.

Target Gene	Name	Direction	Localization	Sequence	Source
*fem^F^*	**femF1**	F	exon 3a	CAACATCTGATGAACTTAAACGG	[16]
	**femF2**	R	exon 6	CTGATTTTTCAATATTTACAGCTAAAACTGTAC	
	femF3	F	exons 2-3a	ACATTTATATTATCTGAAAAATTAG	[17]
	femF4	R	exons 6-3a	GCTTAGATCCTTCTCCCGTTC	
*fem^M^*	**femM1**	F	exon 3b	TGAAGTTAATAACATATTTTTAATTCATCAATGAAG	[16]
	femM2	R	exons 5-4	TGTACCATCTGAAGATTCTAATTTTTCG	
	femM3	F	exon 3a	ATTAGAATCTTCAGATGGTAC	[17]
	femM4	R	exons 4-3b	TATGTAAAATTTAATATATTGCAC	
	femM5	F	exon 3b	GAAGAAAATTTGGACAATGCTG	this study
	**femM6**	R	exon 4	CATGATGCGAATGACTTGATG	
*dsx^F^*	**dsxF1**	F	exon 4	CTATTGGAGCACAGTAGCAAACTTG	[16]
	**dsxF2**	R	exon5	GAAACAATTTTGTTCAAAATAGAATTCC	
*dsx^M^*	**dsxM1**	F	exon 4	CTATTGGAGCACAGTAGCAAACTTG	
	**dsxM2**	R	exon 6	GGCTACGTATGTTTAGGAGGACC	
*rps5*	**rps51**	F	exon 2	CTGCTCACGGGTGATAATCC	this study
	**rps52**	R	exon 3	CTCCTAACTGTACCGGCTCG	
	pJET1.2F	F	---	CGACTCACTATAGGGAGAGCGGC3	Thermo Fisher Scientific
	pJET1.2R	R	---	AAGAACATCGATTTTCCATGGCAG3	

**Table 3:** Thermal cycling conditions used for Real-Time PCR.

Step	Temperature	Duration
UNG activation	50 °C	2 min
UNG inactivation	95 °C	2 min
40 cycles of:		
denaturation	95 °C	15 s
annealing	56 °C for *fem^M^* and *rps5* 58 °C for *fem^F^*, 55 °C for *dsx^M^* and *dsx^F^*	15 s
elongation	68 °C for *fem^M^*, 72 °C for *fem^F^*, *rps5*, *dsx^M^* and *dsx^F^*	1 min

## Data Availability

The original contributions presented in this study are included in the article/Supplementary Materials. Further inquiries can be directed to the corresponding author.

## Supplementary Materials

The following supporting information can be downloaded at: https://www.mdpi.com/article/10.3390/ijms27062756/s1.

## Abbreviations

The following abbreviations are used in this manuscript:

*Apis mellifera*	Western honey bee
C(t)	Quantification cycle
*csd*	Complementary sex determiner
*fem*	Feminizer gene
*fem^F^*	Female-specific feminizer transcript
*fem^M^*	Male-specific feminizer transcript
*dsx*	Doublesex
LOD	Limit of detection
LLOQ	Lower limit of quantification
MIQE	Minimum Information for Publication of Quantitative Real-Time PCR Experiments
RT−	No-reverse transcription control

## Appendix A

**Table A1:** Mean C(t) values of *fem^F^* and *fem^M^* across developmental stages.

Developmental Stage *	Mean C(t)	
	*fem^F^*	*fem^M^*
D-E	39.7	28.7
D-L1	32.7	22.8
D-L2	32.5	21.5
D-L3	35.2	24.9
D-L4	37.9	26.0
D-L5	36.8	22.5
D-PP	33.7	25.2
D-P1	36.2	26.1
D-P2	34.8	22.3
D-P3	37.8	25.2
D-P4	36.0	26.6
D-P5	35.7	27.0
W-E	30.8	32.5
W-L1	25.1	29.8
W-L2	25.1	30.1
W-L3	26.4	31.0
W-L4	26.5	32.4
W-L5	25.4	33.2
W-PP	25.6	31.0
W-P1	23.4	29.8
W-P2	25.4	32.7
W-P3	25.6	30.4
W-P4	24.3	26.2
W-P5	25.5	27.7

* D denotes drones, and W denotes worker bees. E indicates embryos; L1–L5 represent larval stages 1 to 5; PP denotes prepupae; and P1–P5 represent pupal stages 1 to 5.
