# Supplementary material for: Quantitative Analysis of Sex-Specific Feminizer (fem) Transcripts During Honey Bee (Apis mellifera) Development
Source: Int J Mol Sci. 2026 Mar 18;27(6):2756. doi: 10.3390/ijms27062756 (PMC13026495; doi:10.3390/ijms27062756)
Supplement: Supplementary file 1 [file ijms-27-02756-s001.zip › ijms-4185415-supplementary.pdf]

**Table S1.** Amplicon lengths generated by primer pairs used for amplification of *fem*$^F$, *fem*$^M$, and the reference gene *rps5*. Primer orientation (F/R), genomic localization, and expected amplicon lengths are indicated for each primer pair.

| Target gene | Name | Direction | Localization | Sequence | amplicon lengths |
|---|---|---|---|---|---|
| *fem*$^F$ | femF1 | F | exon 3a | CAACATCTGATGAACTTAAACGG | 177 bp |
| | femF2 | R | exon 6 | CTGATTTTTCAATATTTACAGCTAAAACTGTAC | |
| *fem*$^M$ | femM1 | F | exon 3b | TGAAGTTAATAACATATTTTTAATTCATCAATGAAG | 458 bp |
| | femM2 | R | exons 5-4 | TGTACCATCTGAAGATTCTAATTTTTCG | |
| | femM3 | F | exon 3a | ATTAGAATCTTCAGATGGTAC | 912 bp |
| | femM4 | R | exons 4-3b | TATGTAAAATTTAATATATTGCAC | |
| | femM1 | F | exon 3b | TGAAGTTAATAACATATTTTTAATTCATCAATGAAG | 168 bp |
| | femM6 | R | exon 4 | CATGATGCGAATGACTTGATG | |
| *rps5* | rps51 | F | exon 2 | CTGCTCACGGGTGATAATCC | 104 bp |
| | rps52 | R | exon 3 | CTCCTAACTGTACCGGCTCG | |

**Table S2.** Descriptive statistics of Ct values for the reference gene *rps5* across developmental stages. Mean Ct values, standard deviation (SD), minimum and maximum Ct values, and coefficient of variation (CV).

| developmental stage* | Mean C(t) *rps5* | SD | min. | max. | CV % |
|---|---|---|---|---|---|
| D-E | 16.19 | 0.35 | 15.93 | 16.58 | 2.14% |
| D-L1 | 16.75 | 0.35 | 16.45 | 17.14 | 2.10% |
| D-L2 | 16.72 | 0.42 | 16.29 | 17.12 | 2.50% |
| D-L3 | 16.81 | 0.51 | 16.31 | 17.32 | 3.01% |
| D-L4 | 18.96 | 1.43 | 17.63 | 20.47 | 7.54% |
| D-L5 | 18.64 | 0.90 | 17.78 | 19.57 | 4.83% |
| D-PP | 18.73 | 0.26 | 18.44 | 18.94 | 1.41% |
| D-P1 | 18.21 | 1.03 | 17.22 | 19.28 | 5.66% |
| D-P2 | 18.43 | 0.67 | 17.78 | 19.12 | 3.62% |
| D-P3 | 17.98 | 0.70 | 17.26 | 18.66 | 3.88% |
| D-P4 | 19.30 | 0.76 | 18.43 | 19.78 | 3.95% |
| D-P5 | 18.96 | 0.56 | 18.39 | 19.51 | 2.96% |
| W-E | 19.35 | 0.67 | 18.64 | 19.98 | 3.48% |
| W-L1 | 16.54 | 0.32 | 16.19 | 16.82 | 1.95% |
| W-L2 | 16.90 | 0.49 | 16.54 | 17.47 | 2.92% |
| W-L3 | 17.32 | 0.75 | 16.70 | 18.15 | 4.32% |
| W-L4 | 17.23 | 0.48 | 16.72 | 17.68 | 2.81% |
| W-L5 | 17.97 | 0.33 | 17.64 | 18.30 | 1.85% |
| W-PP | 18.64 | 0.45 | 18.30 | 19.16 | 2.43% |
| W-P1 | 17.92 | 0.90 | 16.96 | 18.75 | 5.02% |
| W-P2 | 18.46 | 0.34 | 18.23 | 18.86 | 1.87% |
| W-P3 | 18.21 | 0.67 | 17.76 | 18.98 | 3.71% |
| W-P4 | 19.02 | 0.40 | 18.67 | 19.45 | 2.09% |
| W-P5 | 19.36 | 0.97 | 18.70 | 20.47 | 5.00% |

*D– denotes drones, and W– denotes worker bees. E indicates embryos; L1–L5 represent larval stages 1 to 5; PP denotes prepupae; and P1–P5 represent pupal stages 1 to 5.
